# Supplementary material for: Crystal-confined freestanding ionic liquids for reconfigurable and repairable electronics
Source: Nat Commun. 2019 Feb 1;10:547. doi: 10.1038/s41467-019-08433-5 (PMC6358609; doi:10.1038/s41467-019-08433-5)
Supplement: Supplementary file 1 — Supplementary Information [file 41467_2019_8433_MOESM1_ESM.pdf]

Supplementary Information

Crystal-confined Freestanding Ionic Liquids for Reconfigurable and Repairable Electronics

Naiwei Gao, Yonglin He, Xinglei Tao, Xiao-Qi Xu, Xun Wu, and Yapei Wang *

Department of Chemistry, Renmin University of China, Beijing, 100872, China

E-mail: yapeiwang@ruc.edu.cn

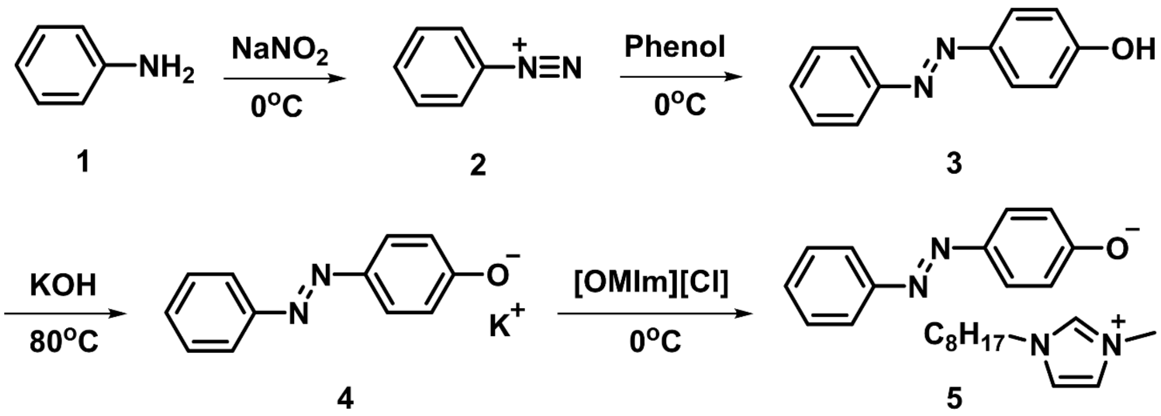

Supplementary Fig. 1 The scheme of the synthetic route of [OMIm]AzoO.

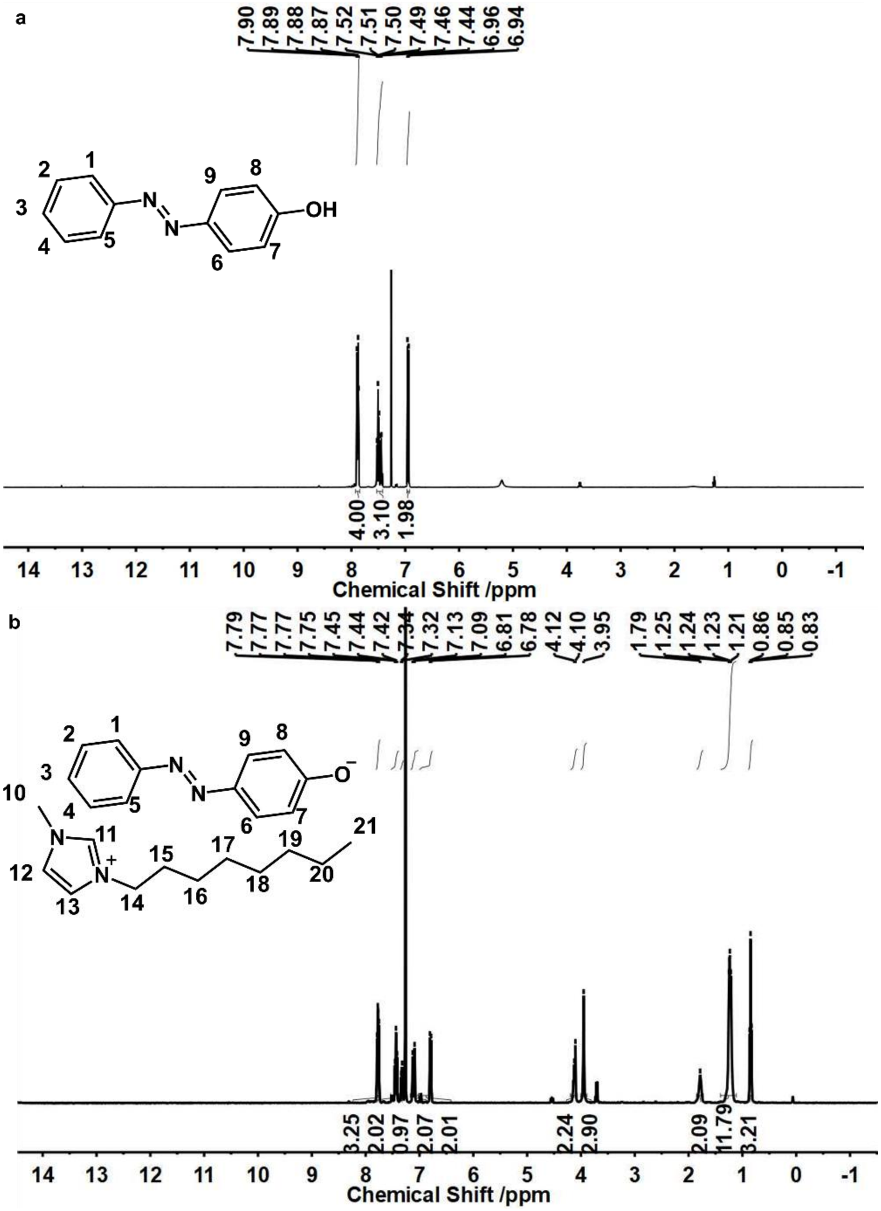

Supplementary Fig. 2 ^{1}H-NMR spectra (400 MHz, $CDCl_3$) of synthesized compounds. **a** (E)-4-(phenyldiazenyl) phenol. **b** 1-octyl-3-methylimidazolium (E)-4-(phenyldiazenyl) phenolate ([OMIm]AzoO).

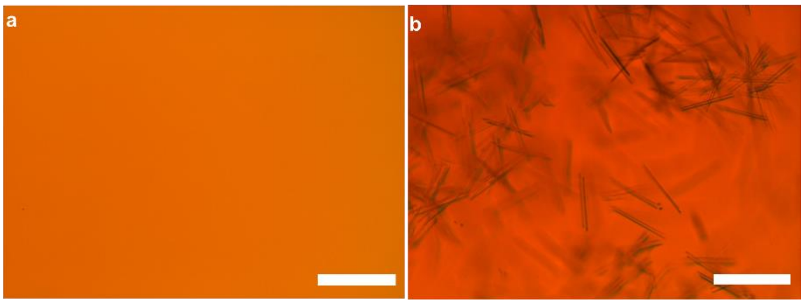

Supplementary Fig. 3 Solubility test of [OMIm]AzoO in [OMIm]PF_6. Microscopic images of CCILs at different addition of [OMIm]AzoO. Scale bar: 200μm. **a** 2 wt.%. **b** 3 wt.%.

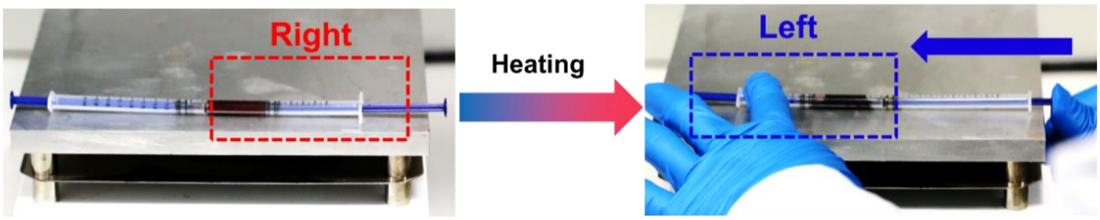

Supplementary Fig. 4 Characterization of reconfigurable performance. Transfer of CCILs with 60 wt.% [OMIm]AzoO from one syringe to another injector at temperature of 70 °C.

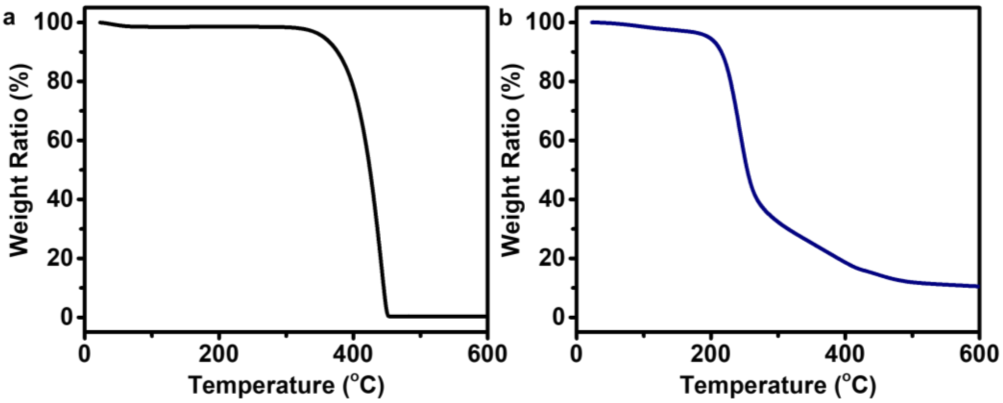

Supplementary Fig. 5 TGA measurement of different ionic liquids. **a** [OMIm]PF_6. **b** [OMIm]AzoO.

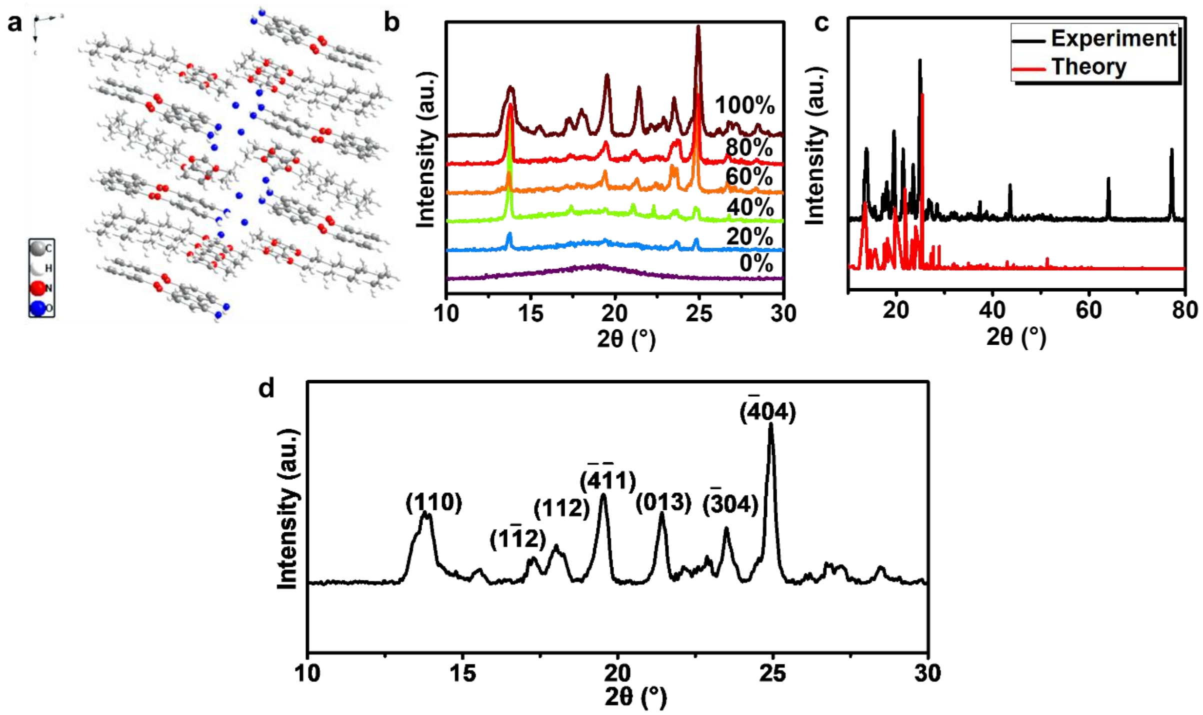

Supplementary Fig. 6 Crystalline characterization of CCILs (crystal-confined ionic liquids). **a** X-ray single diffraction measurement of [OMIm]AzoO. **b** Powder XRD measurement of CCILs at different additions of [OMIm]AzoO. **c** Comparision between the theoretical and experimental XRD data of [OMIm]AzoO. **d** Peak position calibration of XRD of [OMIm]AzoO.

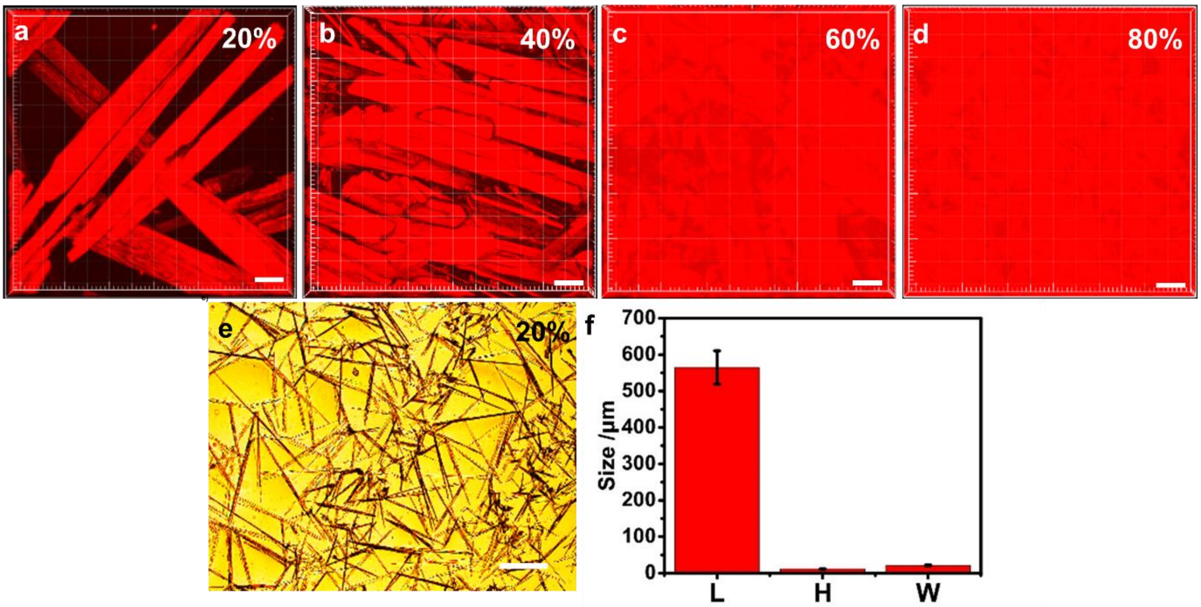

Supplementary Fig. 7 Morphological characterization. Confocal images of CCILs (crystal-confined ionic liquids) with different additions of [OMIm]AzoO: **a** 20 wt.%, **b** 40 wt.%, **c** 60 wt.%, **d** 80 wt.%. Scale bar: 25 μm. **e** Optical image of CCILs with addition of 20 wt.% [OMIm]AzoO. Scale bar: 200 μm. **f** Size statistics of CCILs with addition of 20 wt.% [OMIm]AzoO. L, H, W refer to length, height, and width, respectively.

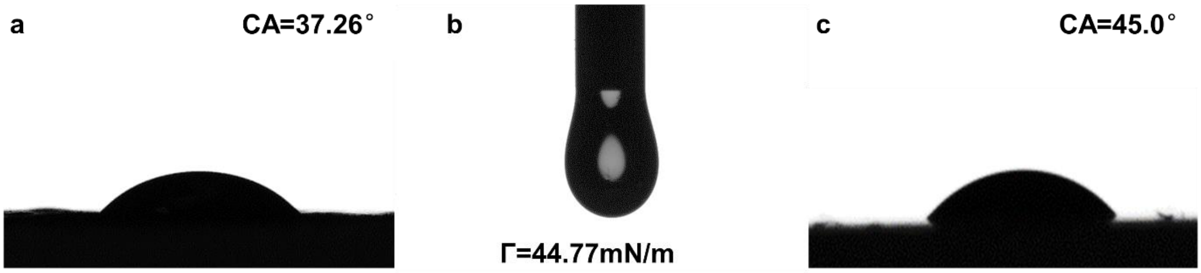

Supplementary Fig. 8 Surface wettability of CCILs (crystal-confined ionic liquids). The measurement of surface wettability of CCILs. **a** Contact angle of the mixture solution of [OMIm]AzoO and [OMIm]PF_6 on [OMIm]AzoO crystals. **b** Surface tension of saturated solution of [OMIm]AzoO in [OMIm]PF_6. **c** Contact angle of the mixture solution of [OMIm]AzoO and [OMIm]PF_6 on glass.

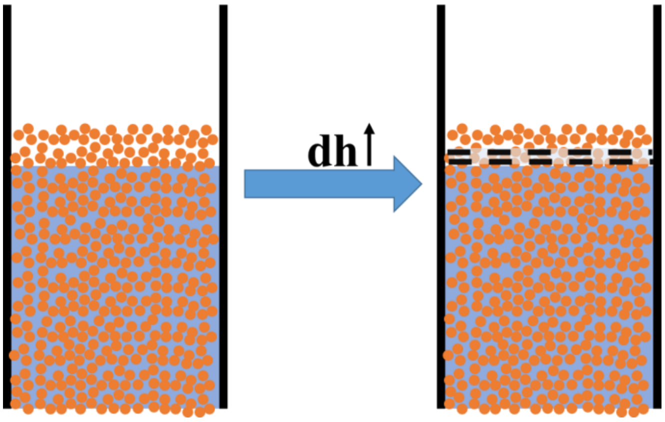

Supplementary Fig. 9 Scheme of equivalent capillary diameter.

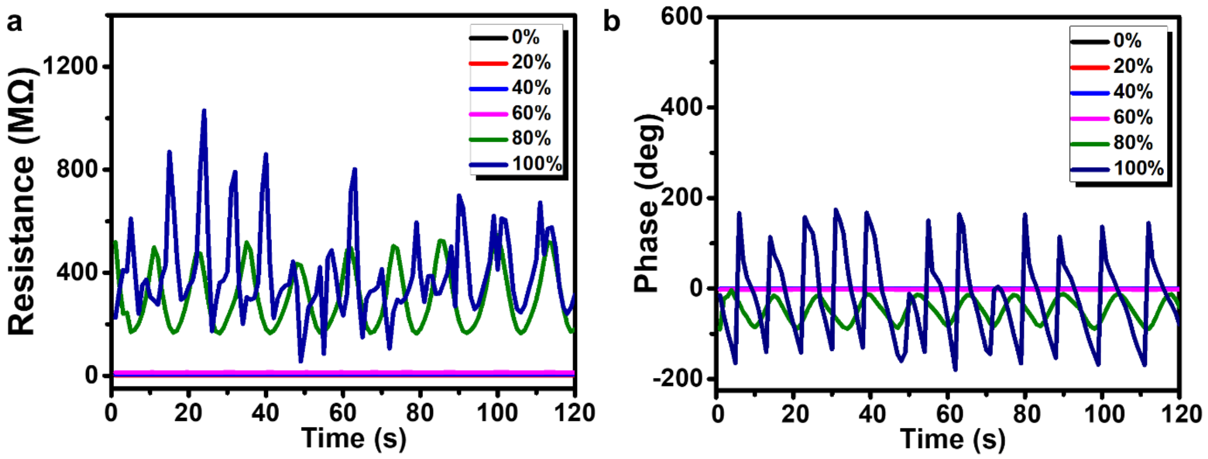

Supplementary Fig. 10 Resistance measurement of CCILs (crystal-confined ionic liquids). Original resistance at different additions of [OMIm]AzoO. **a** Resistance at the addition 0 wt.%, 20 wt.%, 40 wt.%, 60 wt.%, 80 wt.% and 100 wt.%. **b** Phase change at corresponding addition of [OMIm]AzoO.

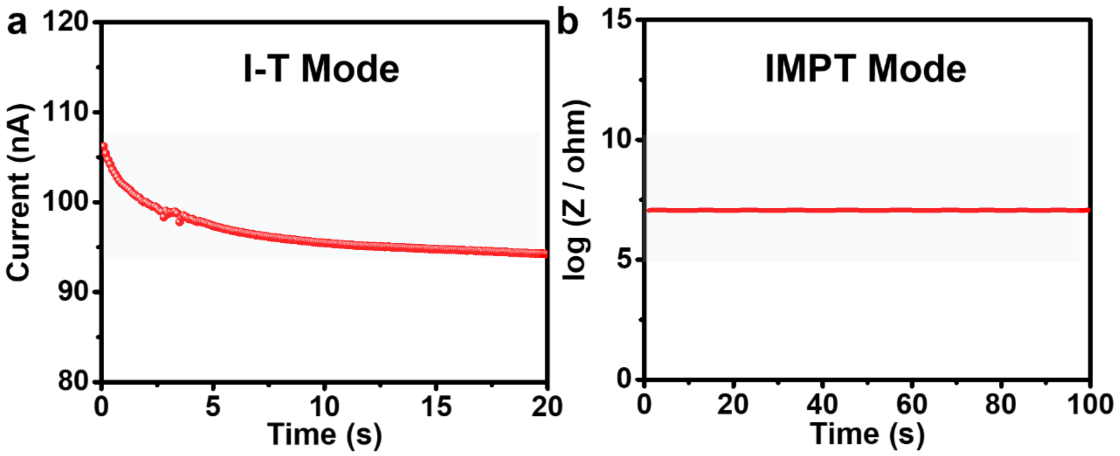

Supplementary Fig. 11 Electrical test by Electrochemical Workstation. **a** Current measurement of CCILs (crystal-confined ionic liquids, 60 wt.%) at 25 ^{o}C in Amperometric i-t curve mode. **b** Resistance measurement of CCILs (60 wt.%) at 25 ^{o}C in Impedance-Time mode.

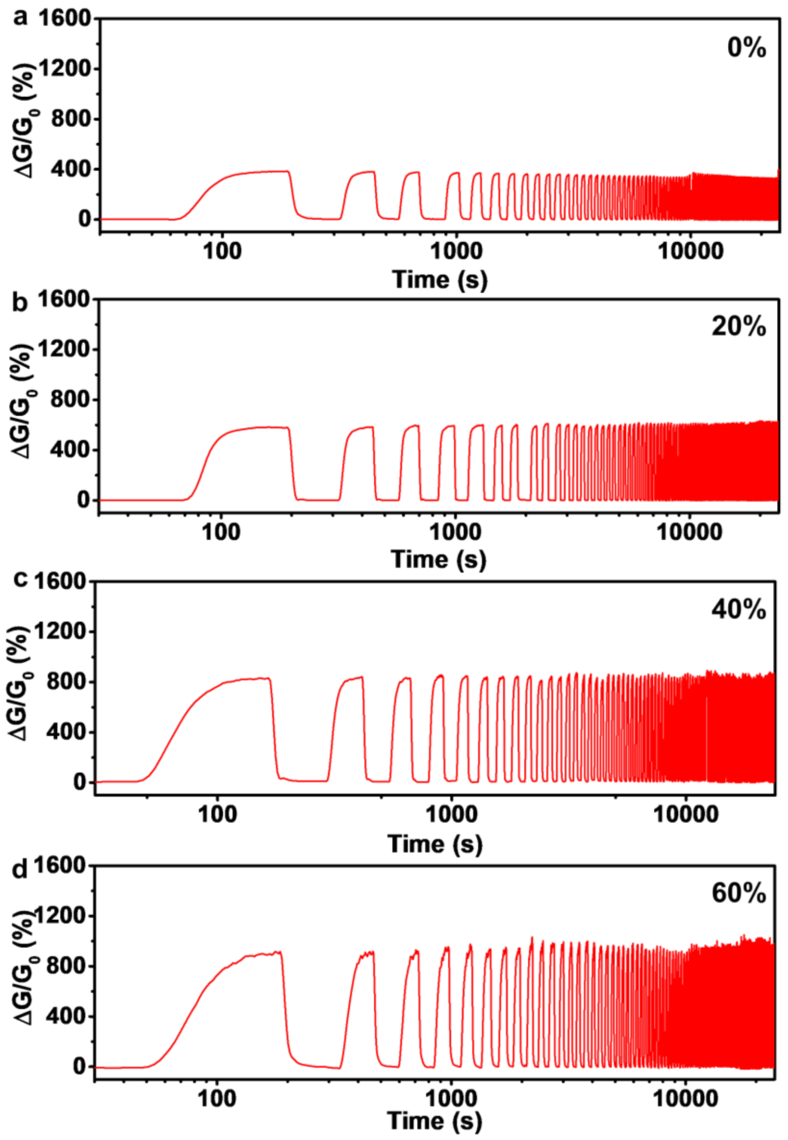

Supplementary Fig. 12 100 cycles of temperature sensing test. **a** CCILs (crystal-confined ionic liquids, 0 wt.% [OMIm]AzoO). **b** CCILs (20 wt.% [OMIm]AzoO). **c** CCILs (40 wt.% [OMIm]AzoO). **d** CCILs (60 wt.% [OMIm]AzoO).

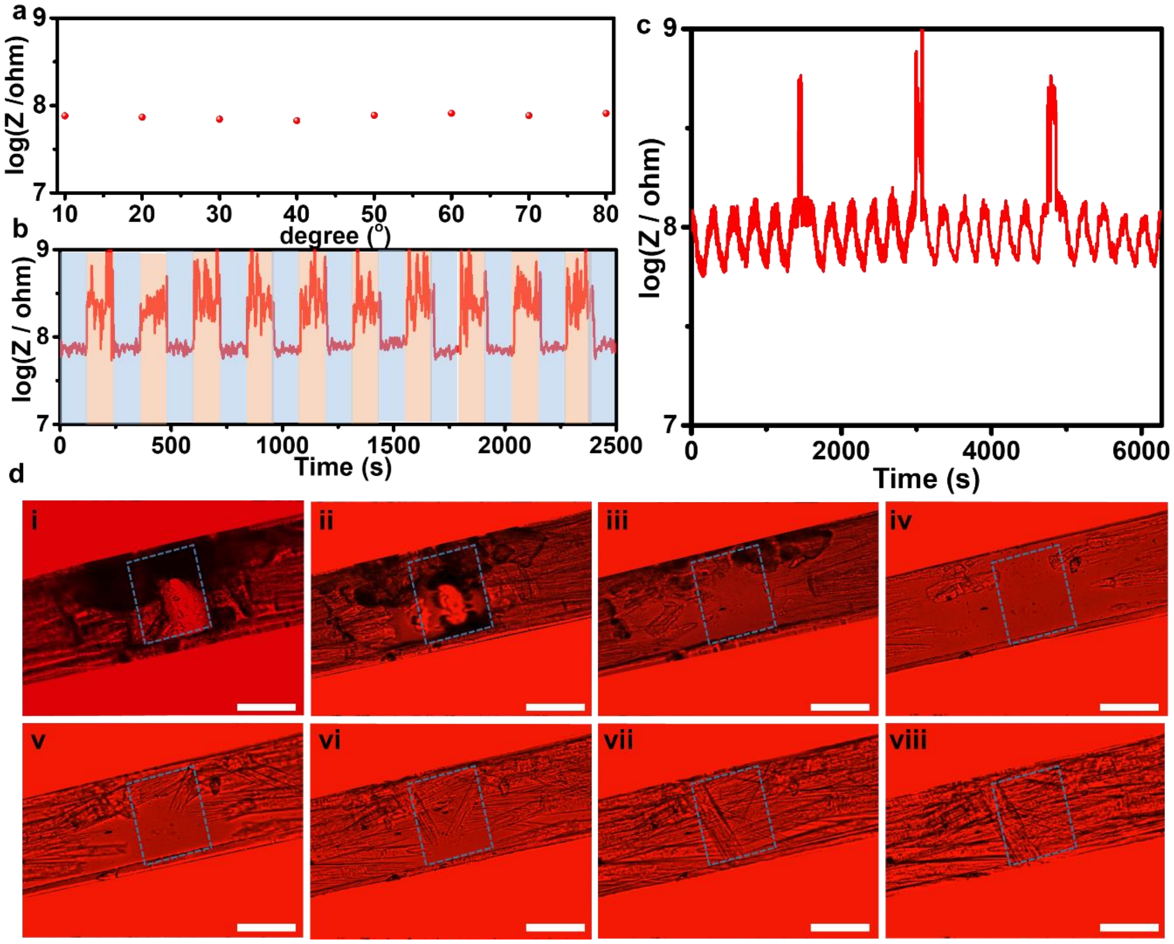

Supplementary Fig. 13 Self-healing measurement of CCILs (crystal-confined ionic liquids, 60 wt.%) at micron-size scale (size: 200 μm), setting the measurement length as 1 cm. **a** Relative resistance at different bending angles. **b** Cycles of electrical repairing after the electrical chip is damaged and repaired for multiple times. (Orange area: Off-state circuits after being cut. Blue area: On-state circuits after repairing.) **c** Cycles of resistance change against temperature change from 25 °C to 40 °C. **d** Microscope image of self-healing process of CCILs (60 wt.%) in a rectangular channel with width of 200 μm. Scale bar: 100 μm.

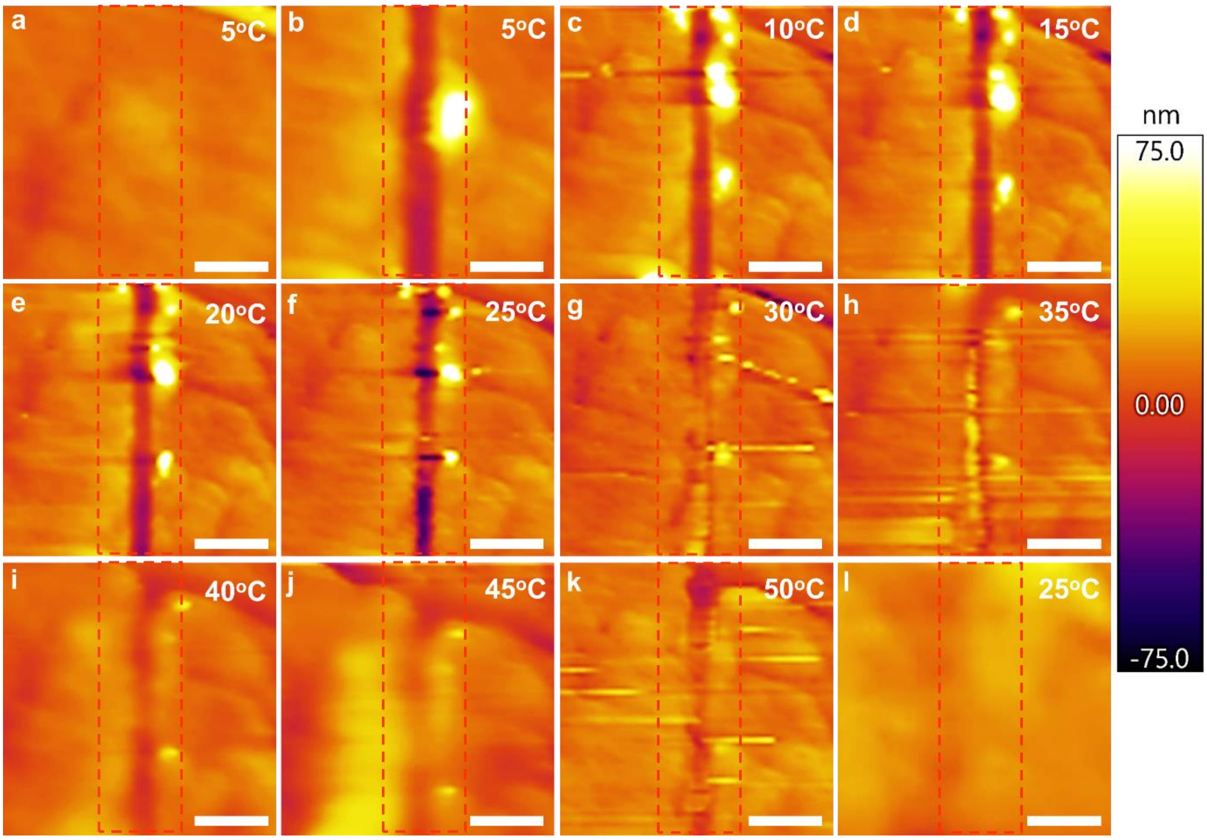

Supplementary Fig. 14 AFM image of self-healing process of CCILs (60 wt.%). AFM image of self-healing process of CCILs (60 wt.%). **a** Original sample (5 ℃). **b** Destroy the surface of sample with AFM tip (5 ℃). **c~k** Raising temperature to 10 ℃, 15 ℃, 20 ℃, 25 ℃, 30 ℃, 35 ℃, 40 ℃, 45 ℃, 50 ℃ gradually. **l** Cooling down to room temperature (25 ℃). Scale bar: 3 μm.

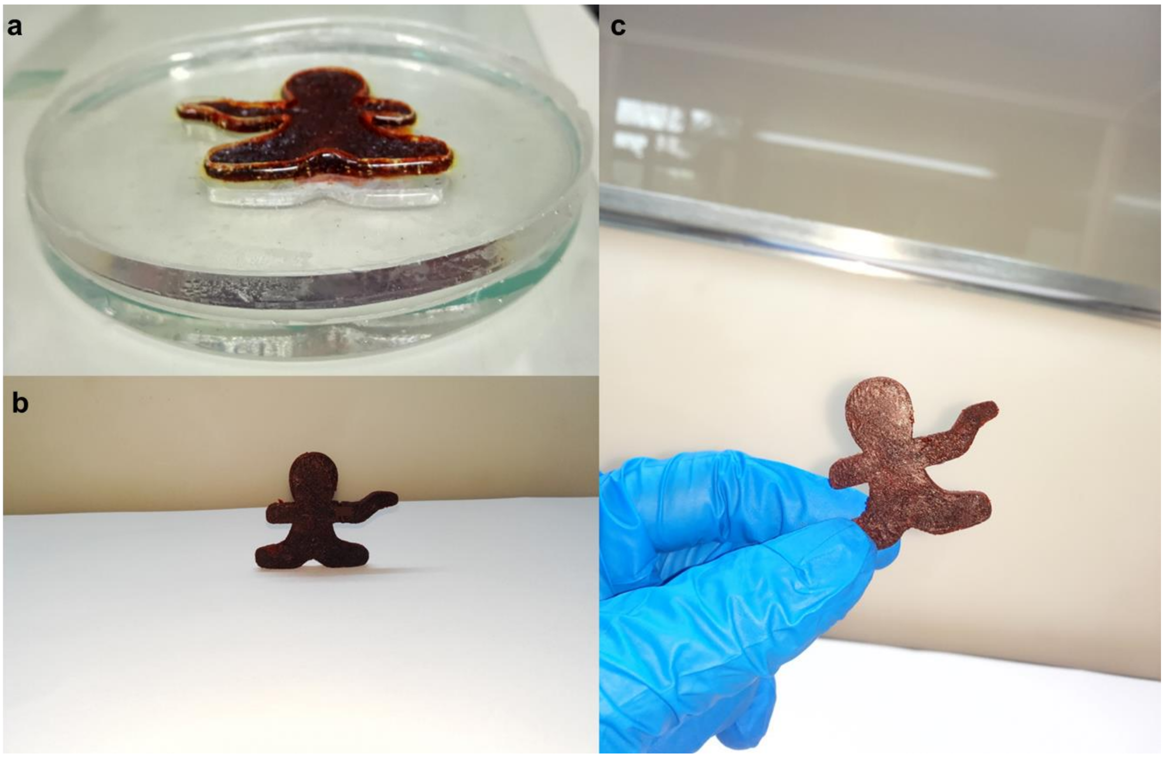

Supplementary Fig. 15 Photos of freestanding CCILs (60 wt.%). **a** CCILs molded in a PDMS template with human-like shape. **b** The human-like CCILs is taken from PDMS mold and placed on the bench. **c** The human-like CCILs is grabbed in hand.

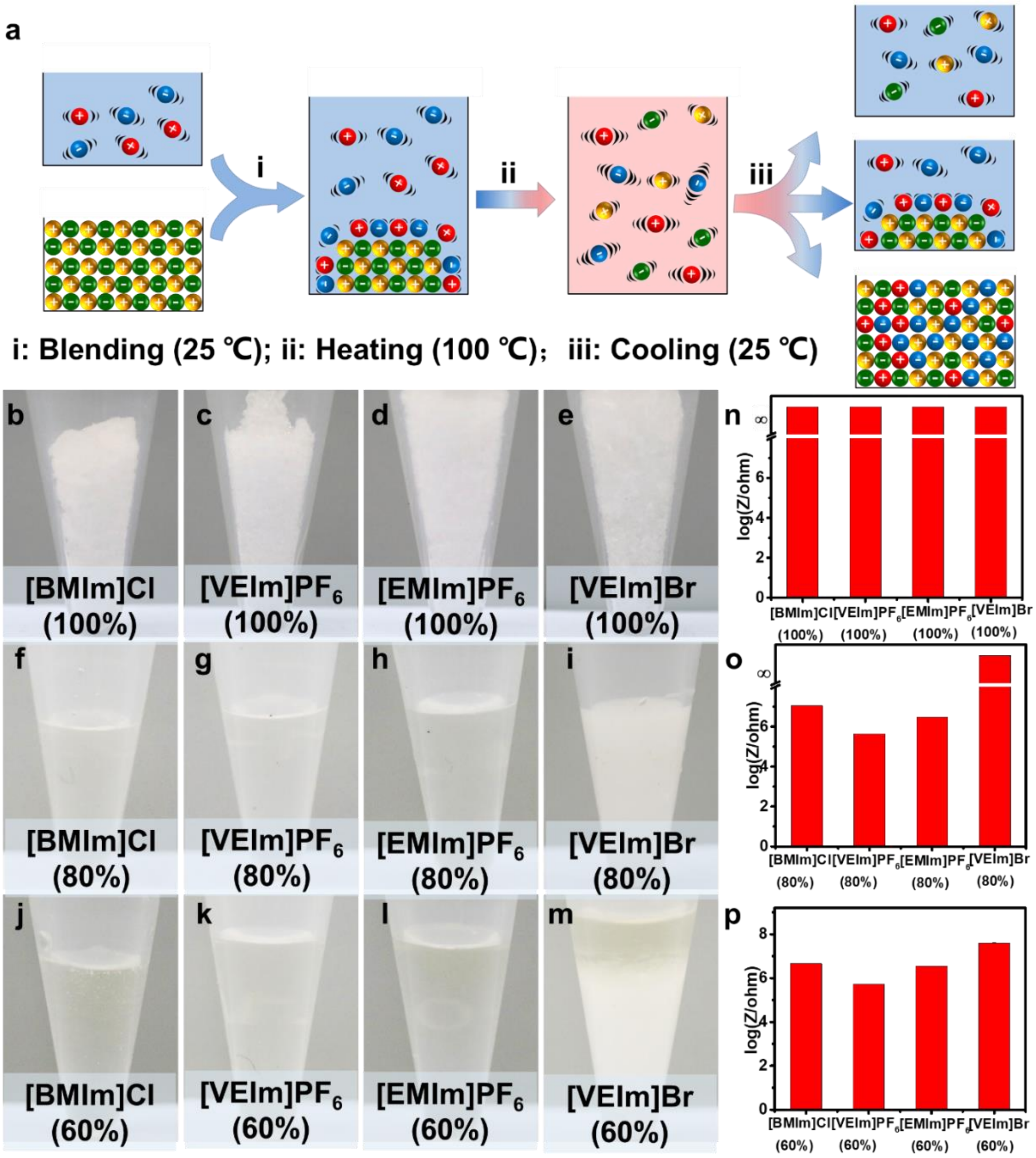

Supplementary Fig. 16 Binary-phase ionic liquids based on different solid ionic liquids. **a** Preparation of CCILs as a complex of [OMIm]PF_6 and solid ionic liquids through a super-saturated solution cooling method. Solid ionic liquids at different ratios of binary-phase ionic liquids as follows: **b** [BMIm]Cl (100 wt.%). **c** [VEIm]PF_6 (100 wt.%). **d** [EMIm]PF_6 (100 wt.%). **e** [VEIm]Br (100 wt.%). **f** [BMIm]Cl (80 wt.%). **g** [VEIm] PF_6 (80 wt.%). **h** [EMIm] PF_6 (80 wt.%). **i** [VEIm] PF_6 (80 wt.%). **j** [BMIm]Cl (60 wt.%). **k** [VEIm] PF_6 (60 wt.%). **l** [EMIm] PF_6 (60 wt.%). **m** [VEIm] PF_6 (60 wt.%). **n** Resistance of Sample b~e. **o** Resistance of Sample f~i. **p** Resistance of Sample j~m.

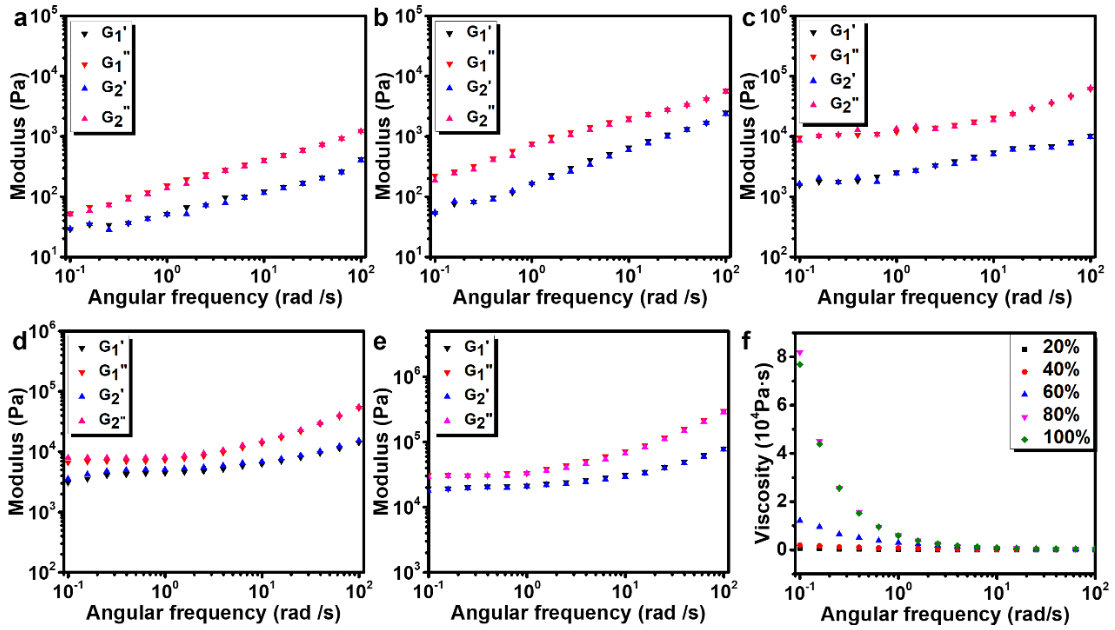

Supplementary Fig. 17 Rheology tests of CCILs (crystal-confined ionic liquids). **a** 20 wt.%. **b** 40 wt.%. **c** 60 wt.%. **d** 80 wt.%. **e** 100 wt.%. **f** Viscosity test of CCILs with different content of [OMIm]AzoO. (Plot 1: before reconfiguration; Plot 2: after reconfiguration)

Supplementary Note 1: Theoretical calculation of CCILs at different states.

When adding [OMIm]AzoO to ILs ([OMIm]PF_6), the CCILs would experience four different states as increasing the amount of [OMIm]AzoO, including solution state, suspended state, loose accumulation state and close-packed state as shown in Fig. 2b. According to microscope images, we could select several representative proportions to calculate these four different states, including 2 wt.% (solution state), 3 wt.% (suspended state), 20 wt.% (loose accumulation state) and close-stacked state (40 wt.%, 60 wt.% and 80 wt.%). There are four different models to calculate the theoretical confinement height of these four states. Corresponding parameters were summarized as follows: ρ_l is the density of [OMIm]PF_6; ρ_s is the density of [OMIm]AzoO; d is the diameter of glass tube; γ_{gl} is the surface tension of saturated solution of [OMIm]AzoO in [OMIm]PF_6; α is the contact angle between saturated solution mentioned above and glass tube; β is the contact angle between saturated solution mentioned above and the crystal [OMIm]AzoO. Accordingly, ρ_l=1.619 kg/m^3, ρ_s=0.814 kg/m^3, d=3 mm, γ_{gl}=0.04477 N/m, $\boldsymbol{\theta}$=37.26^o, β=45°.

For the solution state, the theoretical confinement height can be calculated by classical capillary Supplementary Equation 1.

$$h = \frac{4\gamma_{gl} cos\alpha}{\rho_l g d} \quad (1)$$

For the suspended state, due to the body is still liquid, the traditional capillary equation (Supplementary Equation 2) is still valid. However, the density of liquid needs to be replaced by the average density of CCILs that can be expressed by the $(\rho_L\varphi_L + \rho_S\varphi_S)$. Then the equation is turned as the Supplementary Equation 3.

$$h = \frac{4\gamma_{gl} \cos\beta}{\rho_e g d} \quad (2)$$

$$h = \frac{4\gamma_{gl} \cos\beta}{(\rho_L\varphi_L + \rho_S\varphi_S) g d} \quad (3)$$

In terms of loose accumulation state, the capillary effect of crystals will become the main impact to confine the ionic liquids. Here, we exploited a new method to calculate the theoretical confinement height based on equivalent diameter calculation. More importantly, the sizes of crystals in CCILs (20 wt.%) have been measured by confocal microscopy and optical microscopy so that we could calculate the theoretical confinement height based on the following equation. According to Supplementary Fig. 7f, the length of crystals is 564.5 μm. The width of crystals is 21.3 μm. The height of crystals is 11.4 μm.

As shown in Supplementary Fig. 9, it is difficult to calculate the confinement height through traditional capillary equation. Here, we proposed a solution to avoid the complex calculation based on equivalent capillary equation. Firstly, we could suspect that the liquid level rises with a tiny height of dh. Correspondingly, the volume of liquid would change a tiny value dV_i and the area of liquid would change a tiny value dS_i. Therefore, the new balance force at a certain level could be expressed as:

$$\sum(\Delta P dV_i + (\gamma_{ls} - \gamma_{gs}) dS_i) = 0 \tag{4}$$

Corresponding equation could be deduced as Supplementary Equation 5:

$$\bar{P} = \frac{(\gamma_{gl} - \gamma_{gs}) \sum dS_i}{\sum dV_i} \tag{5}$$

Since the average Laplace pressure $\overline{\mathrm{P}}$ can also be calculated by Supplementary Equation 6:

$$\bar{P} = \frac{4\gamma_{gl} \cos\theta}{d_e} \tag{6}$$

Then we can calculate the equivalent diameter as Supplementary Equation 7:

$$d_e = \frac{4 \sum dV_i}{\sum dS_i} \tag{7}$$

According to

$$\begin{cases} \sum dV_i = V_l \\ \sum dS_i = (m_s + m_l)\sigma \end{cases} \tag{8}$$

Corresponding equation could be obtained as Supplementary Equation 9:

$$d_e = \frac{4\varphi_L}{\rho_s \varphi_S \sigma} \tag{9}$$

Finally, we could get the equation of the theoretical confinement height according to Supplementary Equation 10:

$$h = \frac{\gamma_{gl} \cos\theta \rho_s}{g\rho_L} \times \frac{\varphi_S}{\varphi_L} \times \sigma \tag{10}$$

Here, σ could be calculated based on the completely non-contact crystals as Supplementary Equation 11:

$$\sigma \le \frac{2(WH+WL+HL)}{WHL \times \rho_s} \tag{11}$$

The confinement height could be calculated by Supplementary Equation 12:

$$h_{max} = \frac{2\gamma_{gl} \cos\theta}{g} \times \frac{\rho_S \varphi_S}{\rho_L \varphi_L} \times \frac{WL+WH+HL}{WHL\rho_s} \tag{12}$$

The theoretical confinement height of CCILs (20 wt.%) is 0.3048 m according to Supplementary Equation 12. Since the volume of solid exceeds the volume of liquid in the CCILs (40 wt.%, 60 wt.% and 80 wt.%), the practical sizes of crystals are difficult to be measured and this can also be approved by confocal microscopy and optical microscopy. One important fact is that the confining ability of the CCILs (40 wt.%, 60 wt.% and 80 wt.%) is enhanced in comparison with the CCILs (20 wt.%). When increasing the addition amount of [OMIm]AzoO, there will be more crystals involved in the formation of the capillary system in the presence of the liquids. In this regard, we can calculate the theoretical confinement height by using the sizes of CCILs (20 wt.%). Corresponding theoretical confinement heights are separately 0.8122 m, 1.8275 m and 4.8732 m for the CCILs (40 wt.%, 60 wt.% and 80 wt.%).
