# Supplementary material for: Crystal-confined freestanding ionic liquids for reconfigurable and repairable electronics
Source: Nat Commun. 2019 Feb 1;10:547. doi: 10.1038/s41467-019-08433-5 (PMC6358609; doi:10.1038/s41467-019-08433-5)
Supplement: Supplementary file 3 — Description of Additional Supplementary Files [file 41467_2019_8433_MOESM3_ESM.pdf]

Description of Additional Supplementary Files

File Name: Supplementary Movie 1
Description: The reconfiguration process of crystal-confined ionic liquids. Specially, crystal-confined ionic liquids flow from the upper mold to the bottom mold under thermal treatment.

File Name: Supplementary Movie 2
Description: The self-healing process of an artificial arm made of crystal-confined ionic liquids containing 60 wt.% [OMIm]AzoO. In this special arm, a controlling circuit was implanted to probe mechanical damage and trigger automatic repair.
