# Supplementary material for: Crystal-confined freestanding ionic liquids for reconfigurable and repairable electronics
Source: Nat Commun. 2019 Feb 1;10:547. doi: 10.1038/s41467-019-08433-5 (PMC6358609; doi:10.1038/s41467-019-08433-5)
Supplement: Supplementary file 6 — Source Data [file 41467_2019_8433_MOESM6_ESM.zip › X-ray Data.rtf]

  Table 1.  Crystal data and structure refinement for 1.
Identification code 	1
Empirical formula 	C24 H33 N4 O3
Formula weight 	425.54
Temperature 	150(2) K
Wavelength 	0.71073 Å
Crystal system 	Monoclinic
Space group 	P21/c
Unit cell dimensions	a = 24.520(2) Å	a= 90°.
	b = 6.4191(4) Å	b= 98.057(3)°.
	c = 15.1549(13) Å	g = 90°.
Volume	2361.8(3) Å3
Z	4
Density (calculated)	1.197 Mg/m3
Absorption coefficient	0.080 mm-1
F(000)	916
Crystal size	0.35 x 0.20 x 0.10 mm3
Theta range for data collection	2.52 to 26.41°.
Index ranges	-30<=h<=30, -7<=k<=8, -18<=l<=18
Reflections collected	40303
Independent reflections	4832 [R(int) = 0.1298]
Completeness to theta = 26.41°	99.8 % 
Absorption correction	Semi-empirical from equivalents
Max. and min. transmission	0.9920 and 0.9725
Refinement method	Full-matrix least-squares on F2
Data / restraints / parameters	4832 / 0 / 283
Goodness-of-fit on F2	1.023
Final R indices [I>2sigma(I)]	R1 = 0.0607, wR2 = 0.1516
R indices (all data)	R1 = 0.1077, wR2 = 0.1777
Largest diff. peak and hole	0.553 and -0.465 e.Å-3

 Table 2.  Atomic coordinates  ( x 104) and equivalent  isotropic displacement parameters (Å2x 103)
for 1.  U(eq) is defined as one third of  the trace of the orthogonalized Uij tensor.
________________________________________________________________________________ 
	x	y	z	U(eq)
________________________________________________________________________________  
C(1)	2795(1)	9135(4)	6254(2)	19(1)
C(2)	3668(1)	7959(4)	7030(2)	21(1)
C(3)	3835(1)	10022(4)	7270(2)	19(1)
C(4)	1576(1)	6780(4)	5161(2)	21(1)
C(5)	3166(1)	7524(4)	6539(2)	21(1)
C(6)	2789(1)	8435(4)	8722(2)	23(1)
C(7)	2954(1)	11184(4)	6473(2)	24(1)
C(8)	3457(1)	11629(4)	6971(2)	24(1)
C(9)	2686(1)	6114(4)	8634(2)	23(1)
C(10)	3731(1)	7672(4)	9529(2)	24(1)
C(11)	1243(1)	8430(4)	4816(2)	27(1)
C(12)	2129(1)	5682(4)	8074(2)	26(1)
C(13)	1977(1)	3388(4)	8033(2)	26(1)
C(14)	3425(1)	10819(4)	9721(2)	28(1)
C(15)	1268(1)	686(4)	7385(2)	27(1)
C(16)	1406(1)	2969(4)	7528(2)	28(1)
C(17)	841(1)	4391(4)	4669(2)	31(1)
C(18)	1374(1)	4757(4)	5075(2)	26(1)
C(19)	713(1)	8045(4)	4402(2)	29(1)
C(20)	507(1)	6030(4)	4332(2)	29(1)
C(21)	3932(1)	10656(4)	10192(2)	30(1)
C(22)	4640(1)	7780(5)	10479(2)	31(1)
C(23)	687(1)	314(4)	6915(2)	31(1)
C(24)	538(1)	-1983(5)	6809(2)	37(1)
N(1)	2123(1)	6991(3)	5617(1)	22(1)
N(2)	3305(1)	8934(3)	9309(1)	23(1)
N(3)	2265(1)	8865(3)	5778(1)	21(1)
N(4)	4116(1)	8685(3)	10062(1)	24(1)
O(1)	4312(1)	10412(3)	7737(1)	24(1)
O(3)	5004(1)	7252(3)	8355(1)	28(1)
O(2)	4249(1)	4106(3)	8697(1)	30(1)
________________________________________________________________________________ 
 Table 3.   Bond lengths [Å] and angles [°] for  1.
_____________________________________________________ 
C(1)-C(7) 	1.399(3)
C(1)-C(5) 	1.405(3)
C(1)-N(3) 	1.406(3)
C(2)-C(5) 	1.373(3)
C(2)-C(3) 	1.419(3)
C(2)-H(2) 	0.9500
C(3)-O(1) 	1.304(3)
C(3)-C(8) 	1.418(3)
C(4)-C(11) 	1.394(4)
C(4)-C(18) 	1.390(4)
C(4)-N(1) 	1.425(3)
C(5)-H(5) 	0.9500
C(6)-N(2) 	1.477(3)
C(6)-C(9) 	1.514(4)
C(6)-H(6A) 	0.9900
C(6)-H(6B) 	0.9900
C(7)-C(8) 	1.381(4)
C(7)-H(7) 	0.9500
C(8)-H(8) 	0.9500
C(9)-C(12) 	1.529(3)
C(9)-H(9A) 	0.9900
C(9)-H(9B) 	0.9900
C(10)-N(4) 	1.324(3)
C(10)-N(2) 	1.327(3)
C(10)-H(10) 	0.9500
C(11)-C(19) 	1.382(4)
C(11)-H(11) 	0.9500
C(12)-C(13) 	1.518(4)
C(12)-H(12A) 	0.9900
C(12)-H(12B) 	0.9900
C(13)-C(16) 	1.523(4)
C(13)-H(13A) 	0.9900
C(13)-H(13B) 	0.9900
C(14)-C(21) 	1.347(4)
C(14)-N(2) 	1.374(3)
C(14)-H(14) 	0.9500
C(15)-C(16) 	1.513(4)
C(15)-C(23) 	1.519(4)
C(15)-H(15A) 	0.9900
C(15)-H(15B) 	0.9900
C(16)-H(16A) 	0.9900
C(16)-H(16B) 	0.9900
C(17)-C(18) 	1.386(4)
C(17)-C(20) 	1.385(4)
C(17)-H(17) 	0.9500
C(18)-H(18) 	0.9500
C(19)-C(20) 	1.388(4)
C(19)-H(19) 	0.9500
C(20)-H(20) 	0.9500
C(21)-N(4) 	1.367(3)
C(21)-H(21) 	0.9500
C(22)-N(4) 	1.469(3)
C(22)-H(22A) 	0.9800
C(22)-H(22B) 	0.9800
C(22)-H(22C) 	0.9800
C(23)-C(24) 	1.522(4)
C(23)-H(23A) 	0.9900
C(23)-H(23B) 	0.9900
C(24)-H(24A) 	0.9800
C(24)-H(24B) 	0.9800
C(24)-H(24C) 	0.9800
N(1)-N(3) 	1.266(3)
O(1)-H(1) 	0.8400

C(7)-C(1)-C(5)	118.2(2)
C(7)-C(1)-N(3)	116.4(2)
C(5)-C(1)-N(3)	125.4(2)
C(5)-C(2)-C(3)	122.2(2)
C(5)-C(2)-H(2)	118.9
C(3)-C(2)-H(2)	118.9
O(1)-C(3)-C(8)	122.0(2)
O(1)-C(3)-C(2)	121.5(2)
C(8)-C(3)-C(2)	116.4(2)
C(11)-C(4)-C(18)	119.4(2)
C(11)-C(4)-N(1)	124.9(2)
C(18)-C(4)-N(1)	115.6(2)
C(2)-C(5)-C(1)	120.6(2)
C(2)-C(5)-H(5)	119.7
C(1)-C(5)-H(5)	119.7
N(2)-C(6)-C(9)	112.7(2)
N(2)-C(6)-H(6A)	109.1
C(9)-C(6)-H(6A)	109.1
N(2)-C(6)-H(6B)	109.1
C(9)-C(6)-H(6B)	109.1
H(6A)-C(6)-H(6B)	107.8
C(8)-C(7)-C(1)	121.5(2)
C(8)-C(7)-H(7)	119.3
C(1)-C(7)-H(7)	119.3
C(7)-C(8)-C(3)	121.1(2)
C(7)-C(8)-H(8)	119.4
C(3)-C(8)-H(8)	119.4
C(6)-C(9)-C(12)	110.7(2)
C(6)-C(9)-H(9A)	109.5
C(12)-C(9)-H(9A)	109.5
C(6)-C(9)-H(9B)	109.5
C(12)-C(9)-H(9B)	109.5
H(9A)-C(9)-H(9B)	108.1
N(4)-C(10)-N(2)	108.8(2)
N(4)-C(10)-H(10)	125.6
N(2)-C(10)-H(10)	125.6
C(19)-C(11)-C(4)	119.9(2)
C(19)-C(11)-H(11)	120.0
C(4)-C(11)-H(11)	120.0
C(13)-C(12)-C(9)	113.2(2)
C(13)-C(12)-H(12A)	108.9
C(9)-C(12)-H(12A)	108.9
C(13)-C(12)-H(12B)	108.9
C(9)-C(12)-H(12B)	108.9
H(12A)-C(12)-H(12B)	107.8
C(12)-C(13)-C(16)	113.2(2)
C(12)-C(13)-H(13A)	108.9
C(16)-C(13)-H(13A)	108.9
C(12)-C(13)-H(13B)	108.9
C(16)-C(13)-H(13B)	108.9
H(13A)-C(13)-H(13B)	107.7
C(21)-C(14)-N(2)	107.1(2)
C(21)-C(14)-H(14)	126.4
N(2)-C(14)-H(14)	126.4
C(16)-C(15)-C(23)	113.3(2)
C(16)-C(15)-H(15A)	108.9
C(23)-C(15)-H(15A)	108.9
C(16)-C(15)-H(15B)	108.9
C(23)-C(15)-H(15B)	108.9
H(15A)-C(15)-H(15B)	107.7
C(15)-C(16)-C(13)	114.4(2)
C(15)-C(16)-H(16A)	108.6
C(13)-C(16)-H(16A)	108.6
C(15)-C(16)-H(16B)	108.6
C(13)-C(16)-H(16B)	108.6
H(16A)-C(16)-H(16B)	107.6
C(18)-C(17)-C(20)	120.4(3)
C(18)-C(17)-H(17)	119.8
C(20)-C(17)-H(17)	119.8
C(17)-C(18)-C(4)	120.1(2)
C(17)-C(18)-H(18)	119.9
C(4)-C(18)-H(18)	119.9
C(11)-C(19)-C(20)	120.7(3)
C(11)-C(19)-H(19)	119.7
C(20)-C(19)-H(19)	119.7
C(19)-C(20)-C(17)	119.3(2)
C(19)-C(20)-H(20)	120.3
C(17)-C(20)-H(20)	120.3
C(14)-C(21)-N(4)	107.1(2)
C(14)-C(21)-H(21)	126.4
N(4)-C(21)-H(21)	126.4
N(4)-C(22)-H(22A)	109.5
N(4)-C(22)-H(22B)	109.5
H(22A)-C(22)-H(22B)	109.5
N(4)-C(22)-H(22C)	109.5
H(22A)-C(22)-H(22C)	109.5
H(22B)-C(22)-H(22C)	109.5
C(15)-C(23)-C(24)	113.4(2)
C(15)-C(23)-H(23A)	108.9
C(24)-C(23)-H(23A)	108.9
C(15)-C(23)-H(23B)	108.9
C(24)-C(23)-H(23B)	108.9
H(23A)-C(23)-H(23B)	107.7
C(23)-C(24)-H(24A)	109.5
C(23)-C(24)-H(24B)	109.5
H(24A)-C(24)-H(24B)	109.5
C(23)-C(24)-H(24C)	109.5
H(24A)-C(24)-H(24C)	109.5
H(24B)-C(24)-H(24C)	109.5
N(3)-N(1)-C(4)	113.5(2)
C(10)-N(2)-C(14)	108.2(2)
C(10)-N(2)-C(6)	126.7(2)
C(14)-N(2)-C(6)	125.1(2)
N(1)-N(3)-C(1)	115.2(2)
C(10)-N(4)-C(21)	108.7(2)
C(10)-N(4)-C(22)	125.0(2)
C(21)-N(4)-C(22)	126.3(2)
C(3)-O(1)-H(1)	109.5
_____________________________________________________________ 
Symmetry transformations used to generate equivalent atoms: 
 

 Table 4.   Anisotropic displacement parameters  (Å2x 103) for 1.  The anisotropic
displacement factor exponent takes the form:  -2p2[ h2 a*2U11 + ...  + 2 h k a* b* U12 ]
______________________________________________________________________________ 
	U11	U22 	U33	U23	U13	U12
______________________________________________________________________________ 
C(1)	19(1) 	21(1)	17(1) 	0(1)	5(1) 	-1(1)
C(2)	19(1) 	18(1)	25(1) 	0(1)	4(1) 	2(1)
C(3)	18(1) 	19(1)	20(1) 	-1(1)	5(1) 	-2(1)
C(4)	19(1) 	27(1)	18(1) 	-2(1)	3(1) 	-2(1)
C(5)	24(1) 	17(1)	23(1) 	-3(1)	5(1) 	-1(1)
C(6)	22(1) 	25(1)	23(1) 	2(1)	3(1) 	4(1)
C(7)	25(1) 	19(1)	26(1) 	3(1)	2(1) 	1(1)
C(8)	25(1) 	16(1)	31(1) 	1(1)	2(1) 	-3(1)
C(9)	24(1) 	22(1)	22(1) 	2(1)	4(1) 	3(1)
C(10)	26(1) 	22(1)	25(1) 	-2(1)	3(1) 	3(1)
C(11)	28(2) 	22(1)	31(2) 	1(1)	1(1) 	-4(1)
C(12)	26(1) 	25(1)	26(1) 	3(1)	3(1) 	1(1)
C(13)	26(1) 	26(1)	27(1) 	1(1)	4(1) 	1(1)
C(14)	34(2) 	15(1)	36(2) 	-1(1)	4(1) 	2(1)
C(15)	23(1) 	27(2)	30(2) 	-2(1)	4(1) 	2(1)
C(16)	28(2) 	26(2)	30(2) 	-2(1)	2(1) 	2(1)
C(17)	34(2) 	23(1)	34(2) 	-4(1)	0(1) 	-8(1)
C(18)	26(2) 	23(1)	27(2) 	-3(1)	0(1) 	0(1)
C(19)	27(2) 	27(2)	32(2) 	1(1)	-1(1) 	0(1)
C(20)	23(1) 	35(2)	28(2) 	-3(1)	0(1) 	-5(1)
C(21)	35(2) 	21(1)	35(2) 	-3(1)	6(1) 	-6(1)
C(22)	25(2) 	35(2)	31(2) 	0(1)	0(1) 	3(1)
C(23)	29(2) 	30(2)	33(2) 	-2(1)	1(1) 	1(1)
C(24)	37(2) 	36(2)	36(2) 	-2(1)	2(1) 	-8(1)
N(1)	24(1) 	22(1)	21(1) 	-2(1)	3(1) 	-2(1)
N(2)	25(1) 	21(1)	22(1) 	1(1)	5(1) 	0(1)
N(3)	22(1) 	23(1)	18(1) 	0(1)	3(1) 	-2(1)
N(4)	26(1) 	24(1)	24(1) 	1(1)	5(1) 	0(1)
O(1)	21(1) 	17(1)	35(1) 	-5(1)	0(1) 	-2(1)
O(3)	29(1) 	26(1)	29(1) 	1(1)	2(1) 	2(1)
O(2)	31(1) 	24(1)	37(1) 	-3(1)	6(1) 	2(1)
______________________________________________________________________________ 
 Table 5.   Hydrogen coordinates ( x 104) and isotropic  displacement parameters (Å2x 10 3)
for 1.
________________________________________________________________________________ 
	x 	y 	z 	U(eq)
________________________________________________________________________________ 
 
H(2)	3911	6840	7215	25
H(5)	3069	6121	6391	25
H(6A)	2805	9030	8124	28
H(6B)	2477	9096	8964	28
H(7)	2711	12294	6276	28
H(8)	3551	13036	7114	29
H(9A)	2692	5494	9234	27
H(9B)	2982	5457	8350	27
H(10)	3756	6271	9336	29
H(11)	1379	9816	4867	32
H(12A)	1840	6466	8328	31
H(12B)	2138	6199	7461	31
H(13A)	1993	2847	8648	32
H(13B)	2253	2617	7742	32
H(14)	3194	12013	9680	34
H(15A)	1312	-25	7970	32
H(15B)	1533	55	7027	32
H(16A)	1129	3609	7860	34
H(16B)	1376	3663	6940	34
H(17)	703	3006	4621	37
H(18)	1602	3622	5295	31
H(19)	489	9172	4163	35
H(20)	141	5777	4057	35
H(21)	4124	11712	10548	36
H(22A)	4566	6624	10867	46
H(22B)	4857	8848	10832	46
H(22C)	4846	7264	10014	46
H(23A)	649	969	6318	38
H(23B)	423	1004	7258	38
H(24A)	786	-2664	6444	55
H(24B)	157	-2120	6518	55
H(24C)	576	-2645	7397	55
H(1)	4307	11606	7962	37
________________________________________________________________________________ 
 Table 6.  Torsion angles [°] for 1.
________________________________________________________________ 
 
 
